# *Anopheles gambiae* larvae’s ability to grow and emerge in water containing lethal concentrations of clothianidin, acetamiprid, or imidacloprid is consistent with cross-resistance to neonicotinoids

**DOI:** 10.1186/s13071-024-06188-7

**Published:** 2024-03-01

**Authors:** Marilene Ambadiang, Caroline Fouet, Fred Ashu, Calmes Bouaka, Véronique Penlap-Beng, Colince Kamdem

**Affiliations:** 1 Centre for Research in Infectious Diseases (CRID), Yaoundé, Cameroon; 2 Department of Biochemistry, Faculty of Science, University of Yaoundé 1, Yaoundé, Cameroon; 3 Department of Biological Sciences, The University of Texas at El Paso, El Paso, TX USA

**Keywords:** Pesticides, Neonicotinoids, *Anopheles*, Malaria, Urbanization

## Abstract

**Background:**

For decades, various agrochemicals have been successfully repurposed for mosquito control. However, preexisting resistance caused in larval and adult populations by unintentional pesticide exposure or other cross-resistance mechanisms poses a challenge to the efficacy of this strategy. A better understanding of larval adaptation to the lethal and sublethal effects of residual pesticides in aquatic habitats would provide vital information for assessing the efficacy of repurposed agrochemicals against mosquitoes.

**Methods:**

We reared field-collected mosquito larvae in water containing a concentration of agrochemical causing 100% mortality in susceptible mosquitoes after 24 h (lethal concentration). Using this experimental setup, we tested the effect of lethal concentrations of a pyrrole (chlorfenapyr, 0.10 mg/l), a pyrethroid (deltamethrin, 1.5 mg/l), and three neonicotinoids including imidacloprid (0.075 mg/l), acetamiprid (0.15 mg/l), and clothianidin (0.035 mg/l) on mortality rates, growth, and survival in third-instar larvae of the two sibling species *Anopheles gambiae* and *Anopheles coluzzii* collected from Yaoundé, Cameroon.

**Results:**

We found that *An. gambiae* and *An. coluzzii* larvae were susceptible to chlorfenapyr and were killed within 24 h by a nominal concentration of 0.10 mg/l. Consistent with strong resistance, deltamethrin induced low mortality in both species. Lethal concentrations of acetamiprid, imidacloprid, and clothianidin strongly inhibited survival, growth, and emergence in *An. coluzzii* larvae. By contrast, depending on the active ingredient and the population tested, 5–60% of immature stages of *An. gambiae* were able to grow and emerge in water containing a lethal concentration of neonicotinoids, suggesting cross-resistance to this class of insecticides.

**Conclusions:**

These findings corroborate susceptibility profiles observed in adults and suggest that unintentional pesticide exposure or other cross-resistance processes could contribute to the development of resistance to neonicotinoids in some *Anopheles* populations.

**Graphical Abstract:**

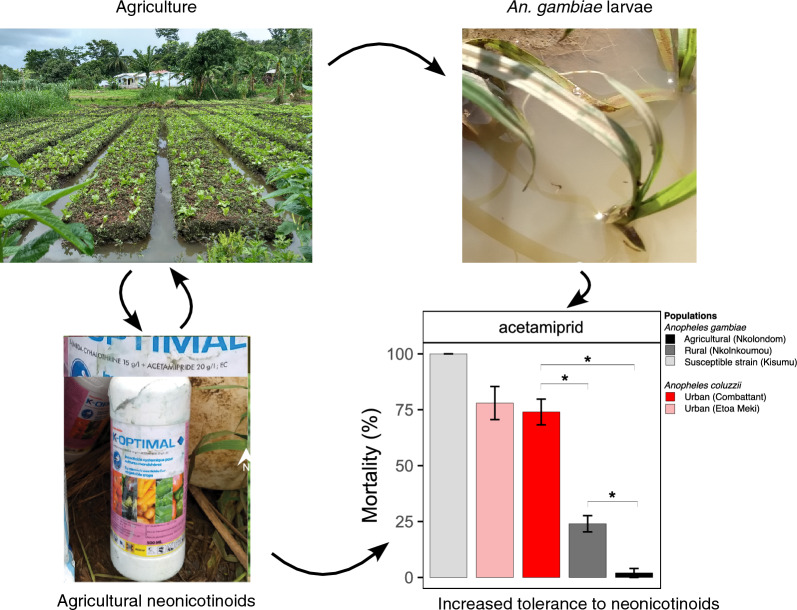

## Background

Prevention based on chemical control of vector populations has contributed to a significant reduction in malaria burden in sub-Saharan Africa over the past two decades [1]. Chemical interventions rely heavily on repurposing of agrochemicals, which provides a rapid and cost-effective approach for screening new active ingredients used against vector populations [2, 3]. To effectively control *Anopheles* populations that are resistant to existing insecticides, dozens of agrochemicals have recently been tested against adult mosquitoes, and some promising candidates have been identified [2, 4–7].

However, cross-resistance—which occurs when resistance to one insecticide reduces susceptibility to another active ingredient, even where the insect has not been exposed to the latter product—can impact the efficacy of some agrochemicals against mosquitoes [8, 9]. For example, prior to the deployment of bed nets impregnated with pyrethroids across Africa, cross-resistance between dichlorodiphenyltrichloroethane (DDT) and pyrethroids as well as agricultural spraying drove resistance detected in some areas in West Africa [10]. Moreover, an important resistant mechanism currently widespread in *Anopheles* mosquitoes involves overexpression of detoxification enzymes that metabolize or prevent the insecticide from reaching its target within the cell [11, 12]. Metabolic resistance due to increased activity of detoxifying enzymes, including esterases (ESTs), glutathione S-transferases (GSTs), and cytochrome P450 monooxygenases (CYPs), is not specific. Therefore, enzymes selected by the exposure to existing insecticides can be used to metabolize new active ingredients.

Classes of agrochemicals, including neonicotinoids, that are widely used for crop protection are particularly exposed to resistance development due to residual pesticides. Additionally, cross-resistance and CYP-based detoxification are both very common mechanisms leading to increased tolerance to neonicotinoids in insect pests [13–17]. Neonicotinoids, a class of eight insecticides, are among the most widely used pesticides in agriculture worldwide [18, 19]. They act as agonists of the nicotinic acetylcholine receptor in the insect’s nervous system and create overstimulation which may result in paralysis and death [19]. Four formulations of clothianidin and one of imidacloprid, two neonicotinoids, are among the prequalified products for indoor residual spraying and space spraying targeting malaria vectors [20–23]. On the other hand, hundreds of formulations of imidacloprid, thiacloprid, thiamethoxam, and acetamiprid are registered for crop protection and are intensively applied in some African countries [24, 25]. These chemicals are highly water-soluble and persistent in the environment, and thus may leach into surface waters [26–28]. In tropical regions, rain and human activities create puddles in farmlands, which become ideal breeding sites for malaria-carrying mosquito species such as *Anopheles gambiae*, *An. arabiensis*, and *An. coluzzii* [29–31]. When these breeding sites are contaminated with pesticides, chronic residual exposure can contribute to pre-adaptation of larval populations to synthetic chemicals [8].

Recent studies assessing the susceptibility of adult *Anopheles* mosquitoes to neonicotinoids suggested that some populations are developing resistance [32–35]. Cross-resistance driven by the spraying of agricultural neonicotinoids is suspected to be the main cause of resistance to clothianidin—an active ingredient which is not used in agriculture and has yet to be applied in public health programs. However, some wild *An. funestus* adult populations that are presumably not exposed to neonicotinoid residues in their larval habitat also display reduced susceptibility to some lethal concentrations of clothianidin [36]. This suggests that even without residual exposure, overexpression of preselected metabolic resistance enzymes may enhance the tolerance to neonicotinoids in some mosquito species [15–17]. Thus, evaluating the baseline susceptibility of wild anopheline populations as well as the impact of residual pesticide exposure and metabolic resistance enzymes on susceptibility could provide critical information for predicting the efficacy of neonicotinoids against anopheline populations.

In Yaoundé, the capital of Cameroon, agricultural activities associated with intensive use of pesticides are pervasive in suburban and rural settings in the outskirts of the city. Mixtures containing neonicotinoids such as acetamiprid, imidacloprid, and thiamethoxam, as well as pyrethroids and fungicides, are frequently sprayed on diverse crops, creating ideal conditions for contamination of surface waters [24, 37]. By contrast, the center of the city provides an island where surface waters and aquatic species are less exposed to pesticide contamination. Two sibling species with contrasting susceptibility to neonicotinoids are sympatric in Yaoundé. *Anopheles coluzzii* adults—the only species present in densely urbanized areas of the city—remains susceptible to neonicotinoids, while its sibling species *An. gambiae*, which occurs in the countryside, has developed resistance [33, 34, 38]. This geographical area thus provides a suitable geographical setting for assessing variability in susceptibility to neonicotinoids between closely related *Anopheles* species.

Most studies evaluating baseline susceptibility to new insecticides focus on testing insecticide-induced mortality in adult mosquitoes [2, 4, 7, 39–42]. Although the level of tolerance among immature stages can provide insights into the role of residual pesticide exposure in resistance selection, this aspect has received little attention. Here, we followed World Health Organization (WHO) guidelines for testing of mosquito larvicides [43] to simultaneously assess the lethal effects (mortality) and some sublethal endpoints (survival, growth, and emergence) in *An. gambiae* and *An. coluzzii*. Larvae were collected from Yaoundé and exposed to lethal concentrations of a pyrrole (chlorfenapyr), a pyrethroid (deltamethrin), or three neonicotinoids (acetamiprid, imidacloprid, and clothianidin). We found that in contrast to *An. coluzzii*, some *An. gambiae* larvae, especially populations from agricultural settings, were able to survive, grow, and emerge in water containing lethal concentrations of neonicotinoids. A stronger adaptation to acetamiprid and imidacloprid—two neonicotinoids that are widely used for crop protection in Cameroon and are known to be persistent in soil–water systems—was observed. We discussed the role of pesticide exposure and of cross-resistance mechanisms in the development of neonicotinoid resistance in immature stages of *Anopheles* mosquitoes.

## Methods

### Study sites

**Fig. 1:**
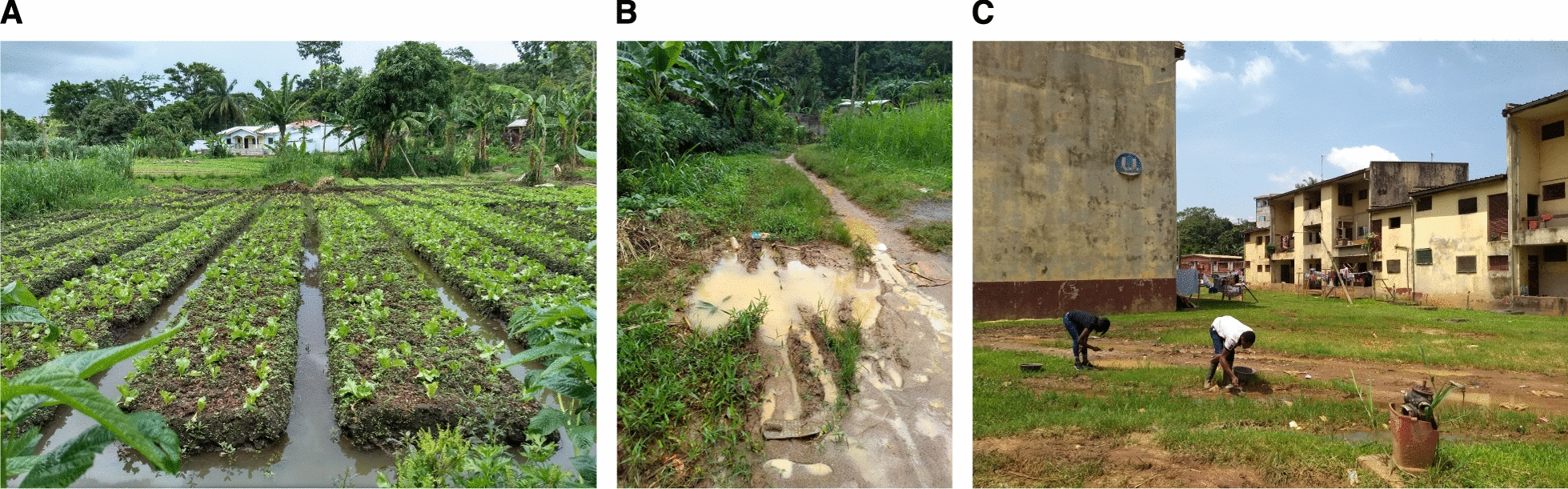
Examples of breeding sites where wild mosquito larvae were sampled. *Anopheles gambiae* larvae were collected from standing water between furrows and ridges in the agricultural area (**A**) or from puddles created by human activities in Nkolnkoumou (suburban area) (**B**). *Anopheles coluzzii* larvae were sampled from human-made puddles in densely urbanized settings (**C**)

The study was carried out in urban and suburban areas of Yaoundé, the capital of Cameroon. Yaoundé lies in the equatorial forest domain of central Africa. Urban areas are surrounded by rural settings characterized by degraded forests. The city experiences four climatic regimes, with two rainy seasons and two dry seasons. Approval to conduct a study in the Center Region (no. 1-140/L/MINSANTE/SG/RDPH-Ce), ethical clearance (no. 1-141/CRERSH/2020), and research permit (no. 000133/MINRESI/B00/C00/C10/C13) were granted by the Ministry of Public Health and the Ministry of Scientific Research and Innovation of Cameroon. We surveyed four sites, including farmland located in the suburbs (Nkolondom, 3°56′43″ N, 11°3′01″ E), two densely urbanized neighborhoods (Etoa Meki, 3°52′53″ N, 11°31′40″ E and Combattant, 3°52′53″ N, 11°31′40″ E), and another suburban area (Nkolnkoumou, 3°52′29″ N, 11°23′2″ E) (Fig. 1). The average distance between sites was 4–5 km.

### Sampling

The study focused on the two sibling species *An. gambiae* and *An. coluzzii*—belonging to the *An. gambiae* complex [*An. gambiae *sensu lato (s.l.)]—which are the most abundant malaria vector populations in Yaoundé [31, 44, 45]. Larvae were collected from locations where extensive surveys have been conducted on *An. gambiae* s.l. populations for several years. The geographical distribution and relative frequencies of the two sibling species *An. gambiae* and *An. coluzzii* in Yaoundé have been well studied [31, 33, 34, 45, 46]. The nominal species *An. gambiae *sensu stricto (hereafter referred to as *An. gambiae*) is the only member of the species complex present in the agricultural site Nkolondom, whereas the most densely urbanized areas (e.g., Combattant and Etoa Meki) harbor exclusively *An. coluzzii* [45–47]. Populations from the suburban neighborhood, Nkolnkoumou, are a mixture of ~ 80% *An. gambiae* and 20% *An. coluzzii* [33, 34, 38]*.* Typical *An. gambiae* s.l. breeding sites were inspected during the rainy seasons in 2022, and larvae were collected using the standard dipping method [48]. Larvae were transported in plastic containers to the insectary where they were identified as species using reference morphological keys [49, 50] and immediately tested in a controlled room (27 °C, 80% relative humidity, light/dark = 12:12 h). In rural and urban settings, larvae thrive in rain-dependent puddles [31]. In the agricultural site, Nkolondom, *An. gambiae* larvae occur in standing waters created by the rain and/or irrigation between furrows and ridges (Fig. 1). Breeding sites contain high concentrations of organic pollutants in some densely urbanized settings and are likely contaminated with pesticide residues in agricultural areas in Yaoundé [31, 45].

### Insecticides

We selected and tested five insecticides with different levels of application in agriculture and in public health in Cameroon. This included two neonicotinoids (acetamiprid and imidacloprid), which are known to be highly persistent in soil and surface waters and are among the most widely used pesticides in Cameroon [27, 37]. Imidacloprid is also the active ingredient in a formulation prequalified for indoor/outdoor space spraying [23]. A third neonicotinoid (clothianidin) which is also persistent but is not currently used in agriculture or in public health in Cameroon was tested [24, 37, 51]. Clothianidin is the active ingredient in four new indoor residual spraying formulations [23]. We also tested chlorfenapyr, a pyrrole, and deltamethrin, a pyrethroid. Chlorfenapyr is used in a new generation of long-lasting insecticidal nets that have not yet been officially deployed on a large scale in Cameroon [23, 52]. Deltamethrin is a pyrethroid used in long-lasting insecticidal nets and indoor residual spraying for two decades which can also be found in a variety of crop-protection formulations registered in Cameroon [10, 37]. The following commercial formulations were tested: chlorfenapyr (Pestanal, analytical standard, Sigma-Aldrich), acetamiprid (Aceplant 40EC, 40 g/l, emulsifiable concentrate, JACO, Yaoundé, Cameroon), imidacloprid (Plantima 30SC, 30 g/l, concentrated suspension, JACO, Yaoundé, Cameroon), clothianidin (Pestanal, analytical standard, Sigma-Aldrich), and deltamethrin (Decis 25EC, 25 g/l, emulsifiable concentrate, Bayer Cropscience S.L., Valencia, Spain). Stock solutions were prepared by diluting the formulation in absolute ethanol or distilled water.

### Lethal concentration determination

To assess the lethal and sublethal effects of each insecticide, we reared field-collected larvae in water containing a nominal concentration of the insecticide causing 100% mortality in a susceptible strain within 24 h (lethal concentration), and we measured mortality as well as some life history parameters. The mosquito life cycle includes four larval stages prior to pupation and emergence of a male or female adult [48]. The four stages comprise the first (L1), second (L2), third (L3), and fourth (L4) instars and can last between 1 and 2 weeks depending on the mosquito species, the feeding regime, and the environmental conditions. We monitored mortality and life table parameters from L3 to emergence. Third-instar larvae of *Anopheles* are abundant in the wild, are easy to identify morphologically, and typically complete transformation into L4, pupation, and emergence within approximately 7 days.

The lethal concentration used for each insecticide was determined as follows: we exposed L3 larvae of the susceptible strain *An. gambiae* Kisumu to increasing concentrations of the insecticide, starting from 0.001 mg/l, and we retained the lowest concentration causing 100% mortality within 24 h. *Anopheles gambiae* Kisumu, established as a laboratory strain since 1975, is susceptible to common classes of insecticides used in mosquito control, including pyrethroids, organophosphates, carbamates, and organochlorines. Four replicates were tested for each insecticide dose in addition to a control without insecticide (i.e., containing only water). This experimental setup based on WHO guidelines for laboratory and field testing of mosquito larvicides has been successfully used to evaluate survival in larvae of the yellow fever mosquito *Aedes aegypti* reared in water containing a pesticide [43, 53]. Batches of 25 larvae were placed in 500-ml plastic trays filled with 200 ml of borehole water containing the desired concentration of insecticide and covered with a net. Borehole water was used for routine maintenance of *Anopheles* mosquito colonies in the insectary and was thus preferred to distilled water. Water was collected from a borehole located in Odza, Yaoundé (3°47′60.0″N, 11°31′60.0″E). To minimize variability, we collected a large quantity of water that was used for all the experiments.

### Biological activity testing

Larvae were collected from the field in the morning and brought to the insectary. Third-instar larvae were sorted immediately and rinsed in a tray containing borehole water before being transferred into test trays. Four replicates of 25 larvae were tested in 500-ml plastic trays that were filled with 200 ml borehole water containing the lethal concentration of the insecticide detected as described above. For each insecticide, a control test was conducted concomitantly by rearing two batches of 25 larvae in water alone. Water was not changed throughout the experiment, and 10 mg of TetraMin^®^ fish food was added to each tray daily. Every 24 h, the number of L3, L4, pupae, and adults was counted in each tray. Larvae were considered dead if they were unable to move when touched with a dropper. Dead larvae were removed from the test containers and were not replaced. Adults were also removed using a mouth aspirator. Survival, growth, pupation, and emergence were assessed daily for 7 days, which was sufficient for L3 larvae to reach the adult stage.

### Data analysis

The lethal endpoint of insecticide exposure was assessed using the mortality rate at 24 h. The sublethal effects of the different insecticides were addressed using survival probability as well as L4 transformation rate, pupation rate, and emergence rate. L4 transformation rate was defined as the percentage of L3 larvae transformed into L4 at a given time point. Pupation rate represented the percentage of L3 that made it to the pupa stage, and emergence rate the percentage of L3 that reached the adult stage. Mean and standard error, computed with the packages *plyr* and *ggplot2* in R (version 4.2.), were used to estimate L4 transformation rate, pupation rate, and emergence rate at 24-h intervals. Fisher’s exact test was applied for pairwise comparisons between populations. The ratio between the number of dead larvae and the initial number of individuals was calculated every 24 h and provided an estimate of survival probability. Kaplan–Meier survival curves were plotted using the packages *ggplot 2*, *ggfortify*, and *survival* in R [54]. Larvae that reached the adult stage were treated as censored data. Confidence intervals were computed for the four replicates, and a log-rank test was used to determine if survival was significantly different between treatments and between populations.

## Results

### 24-hour lethal toxicity

**Fig. 2:**
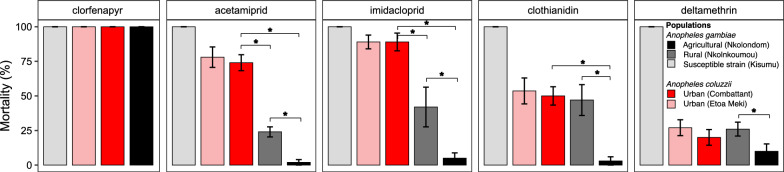
24-h mortality of mosquito larvae reared in water containing a lethal concentration of agrochemical. A concentration killing 100% of larvae from the susceptible strain *An. gambiae* Kisumu was used to test field-collected individuals. Wild populations were susceptible to chlorfenapyr but showed varying levels of tolerance to four other agrochemicals. Mortality values were lowest in *An. gambiae* larvae collected from an agricultural area (Nkolondom). Error bars represent the standard error of the mean. *Fisher’s exact test, *P* < 0.05

We studied the lethal and sublethal effects of agrochemicals on field-collected *Anopheles* larvae using lethal concentrations of 0.10 mg/l for chlorfenapyr, 0.035 mg/l for clothianidin, 0.075 mg/l for imidacloprid, 0.15 mg/l for acetamiprid, and 1.5 mg/l for deltamethrin. The pyrrole chlorfenapyr was the most toxic among the five insecticides tested. All field-collected larvae were fully susceptible to this insecticide and were killed within 24 h (Fig. 2). By contrast, neither neonicotinoids nor deltamethrin were able to cause 100% mortality within 24 h in wild larvae. In water containing neonicotinoids or deltamethrin, mortality was significantly lower in all field-collected larvae compared to the susceptible strain *An. gambiae* Kisumu (Fisher’s exact test, *P* < 0.05). Mortality rates were typically below 40% in deltamethrin. Pairwise comparisons also revealed that *An. gambiae* larvae from Nkolondom displayed lower mortality to deltamethrin compared to the sister population (Nkolnkoumou, Fisher’s exact test, *P* = 0.005) and to immature stages of *An. coluzzii* collected from Etoa Meki (Fisher’s exact test, *P* = 0.003). Susceptibility to neonicotinoids varied strongly between species and geographical areas. *Anopheles coluzzii* larvae collected from the two urban neighborhoods, Combattant and Etoa Meki, were more susceptible, with mortality rates between 70 and 80%, except clothianidin, for which only about 50% of larvae were killed after 24 h. Both *An. coluzzii* populations displayed comparable mortality in clothianidin (Fisher’s exact test, *P* = 0.87), in acetamiprid (*P* = 0.62), and in imidacloprid (*P* = 1) (Fig. 2). Conversely, *An. gambiae* larvae were substantially more tolerant to neonicotinoids, especially individuals collected from the farm (Nkolondom), which displayed less than 10% mortality in water containing the insecticide. Mortality rates were also significantly lower among larvae from Nkolondom compared to conspecific individuals collected from Nkolnkoumou in acetamiprid (Fisher’s exact test, *P* < 0.0001), in clothianidin (*P* < 0.0001), and in imidacloprid (*P* < 0.0001).

### Survival probability

**Fig. 3:**
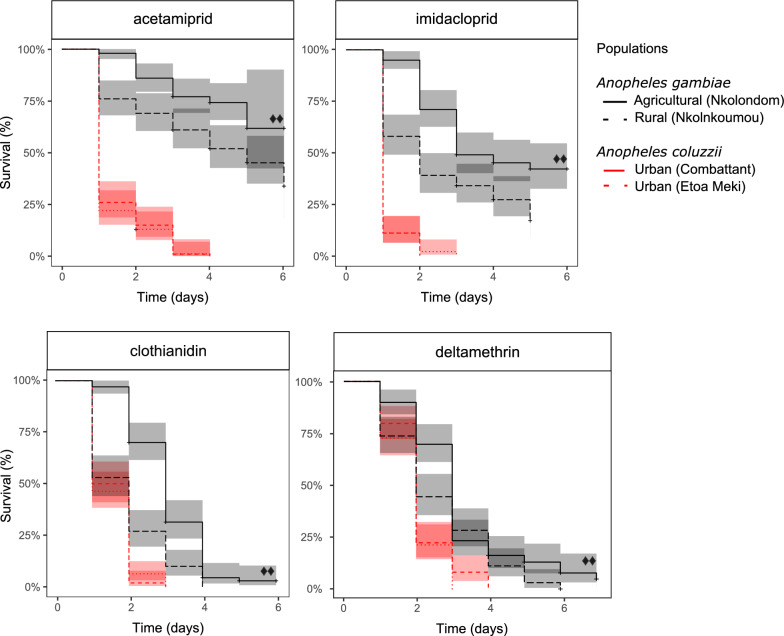
Kaplan–Meier survival curves (lines) with 95% confidence intervals (colored bands) of L3 larvae reared in water containing a lethal concentration of pesticide. Survival probabilities of four wild populations were compared under controlled laboratory conditions. + indicates emergence that occurred before the end of the experiment and was treated as censored data. Filled diamonds: log-rank test indicates *An. gambiae* (Nkolondom) has higher survival compared to *An. gambiae* (Nkolnkoumou) and to *An. coluzzii* (Etoa Meki)

Rearing field-collected larvae in water containing a lethal concentration of agrochemical affected their survival probability as revealed by Kaplan–Meier survival curves (Fig. 3). Larvae that survived lethal toxicity within the first 24 h were monitored for 7 days. Survival within 7 days mirrored the results of 24-h mortality and confirmed high fitness of some *Anopheles* larval populations in water containing a lethal concentration of a neonicotinoid of deltamethrin. There was no significant difference in survival between the two *An. coluzzii* larval populations tested in deltamethrin or in neonicotinoids (*P* > 0.05, log-rank test) (Fig. 3). In deltamethrin, *An. gambiae* larvae from Nkolondom had a higher survival probability compared to immature stages of *An. coluzzii* from Etoa Meki (log-rank test, *P* < 0.0001) and to those of *An. gambiae* from Nkolnkoumou (*P* = 0.005). When field-collected larvae were reared in water containing a neonicotinoid, differences in survival were more pronounced between the sibling species *An. gambiae* and *An. coluzzii*. Notably, *An. gambiae* larvae from Nkolondom had significantly higher survival rates in acetamiprid (log-rank test, *P* < 0.0001) and in imidacloprid (*P* < 0.0001) compared to the urban population of *An. coluzzii* (Etoa Meki). In water containing clothianidin, immature stages from the agricultural site (Nkolondom) displayed higher survival compared to conspecific (Nkolnkoumou, log-rank test, *P* = 3.13E−07) and to heterospecific (Etoa Meki, *P* < 0.0001) larval populations. Survival of 100% was obtained in all control experiments without insecticide.

### L4 transformation rate

**Fig. 4:**
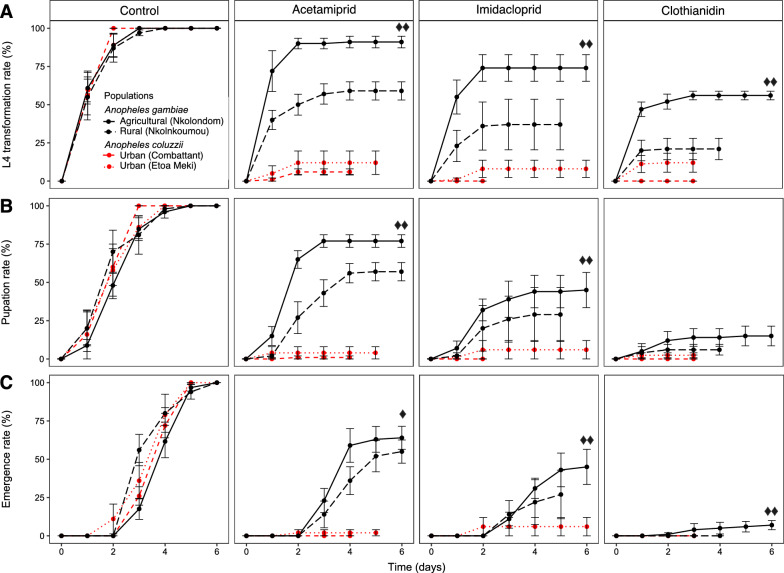
Sublethal effects of three neonicotinoid insecticides on life table parameters in *Anopheles* larvae. Larvae that survived 24-h lethal toxicity were monitored for 7 days under standard laboratory conditions while measuring the rate of transformation of third instars (L3) into fourth instars (L4) (**A**), pupation rate (**B**), and emergence rate (**C**). Controls were larvae reared in water without insecticide. Vertical bars represent the standard error of the mean. Double diamonds: Fisher’s exact test (*P* < 0.05) indicates a significant difference between *An. gambiae* (Nkolondom) and *An. gambiae* (Nkolnkoumou), and between *An. gambiae* (Nkolondom) and *An. coluzzii* (Etoa Meki). Single diamond: Fisher’s exact test (*P* < 0.05) indicates a significant difference between *An. gambiae* (Nkolondom) and *An. coluzzii* (Etoa Meki)

Focusing on neonicotinoids, we used life table analysis to further dissect some of the sublethal adjustments that likely contribute to the development of resistance to repurposed agrochemicals in *Anopheles* larvae. We started by comparing L4 transformation rate between field-collected larval populations. Consistent with their high tolerance revealed by mortality and survival analyses, a large proportion of *An. gambiae* L3 larvae were able to turn into L4 in water containing a lethal concentration of neonicotinoid (Fig. 4a). The highest transformation rate was observed in populations from Nkolondom: 90 ± 3% in water containing acetamiprid followed by 75 ± 9% in imidacloprid within 2 days and 60 ± 3% in clothianidin after 3 days. Similarly, in larvae collected from Nkolnkoumou, a semi-rural site harboring ~ 80% *An. gambiae*, transformation rates varied from 60 ± 6% for acetamiprid, to 35 ± 16% and 23 ± 7% for imidacloprid and clothianidin, respectively, after 2–4 days. On the other hand, *An. coluzzii* larvae were strongly inhibited in water treated with a neonicotinoid, leading to very low transformation rates between 0 and 10% among immature stages from Etoa Meki or Combattant. No difference in transformation rate was observed between both *An. coluzzii* populations (Fisher’s exact test, *P* = 0.216). Conversely, the rate of transformation from L3 to L4 in acetamiprid, imidacloprid, or clothianidin was eightfold (*P* < 0.0001), ninefold (*P* < 0.0001), and fourfold (*P* < 0.0001) higher, respectively, in *An. gambiae* larvae from Nkolondom compared to immature stages of *An. coluzzii* samples from Etoa Meki. Similarly, *An. gambiae* larvae from the agricultural site had higher transformation rates in neonicotinoids compared to those collected from Nkolnkoumou in the suburban area (Fisher’s exact test, *P* < 0.05) (Fig. 4a). In control tests (without insecticide), transformation rates were 100% after 2–3 days, and there was no significant difference between the four field populations (*P* > 0.05).

### Pupation rate

Pupation rate was consistent with L4 transformation and highlighted the low susceptibility of agricultural and semi-rural populations of *An. gambiae* to neonicotinoids (Fig. 4b). The agricultural population of *An. gambiae* (Nkolondom) had the highest pupation rates, 77.5 ± 4% in water containing acetamiprid, and 45 ± 11% in imidacloprid. The exotic insecticide, clothianidin, at a nominal concentration of 0.035 mg/l, had the strongest inhibitory effect among neonicotinoids, although 15–20% of *An. gambiae* larvae completed pupation. *Anopheles coluzzii* larvae had low pupation rates with less than 5% pupae obtained in any neonicotinoid tested. Notably, none of the *An. coluzzii* populations were able to pupate in clothianidin. Pupation rate was significantly higher in larvae from Nkolondom versus Nkolnkoumou in acetamiprid (Fisher’s exact test, *P* = 0.004) and in imidacloprid (*P* = 2.77E−2) but not in clothianidin (*P* = 0.063). All larvae reached the pupa stage in all the control tests within 72 h, and there was no difference between larval populations reared in water without insecticide (*P* > 0.05).

### Emergence rate

All (100%) *An. gambiae* and *An. coluzzii* pupae emerged in control water without insecticide between the first and the sixth day in standard laboratory conditions. The emergence rate of *An. coluzzii* larvae was 0%, 2%, and 4% in water containing clothianidin, acetamiprid, or imidacloprid, respectively, after 6 days (Fig. 4c). Meanwhile, at least half of *An. gambiae* L3 larvae tested emerged between 2 and 7 days in acetamiprid and in imidacloprid. Only *An. gambiae* larvae collected from Nkolondom were able to emerge in clothianidin, albeit at a lower rate (7 ± 3%) compared to the other neonicotinoids. The emergence rate after 6 days in acetamiprid and in imidacloprid was 32-fold (Fisher’s exact test, *P* < 0.0001) and eightfold (*P* < 0.0001) higher, respectively, in *An. gambiae* larvae (Nkolondom) compared to juveniles of *An. coluzzii* (Etoa Meki). Within the *An. gambiae* species, larvae from the farm emerged at a higher rate compared to individuals form Nkolnkoumou in imidacloprid (*P* = 0.012) and in clothianidin (*P* = 0.014), but not in acetamiprid (*P* = 0.249).

## Discussion

In this study, we have used standard bioassays to simultaneously assess the lethal and residual effects of pesticide exposure in mosquito larvae. We found that monitoring some sublethal changes in a controlled aquatic environment provides critical information on the current level of tolerance in larval populations. We have hypothesized that larval susceptibility profiles could provide complementary information for predicting the efficacy of repurposed agrochemicals against adult mosquitoes. However, the susceptibility of larvae to an active ingredient does not necessarily reflect that of adults and vice versa [43, 55]. Immature stages of insect pests can be more susceptible or more resistant to some insecticides than adults. Nevertheless, by comparing larval tolerance detected in this study to the susceptibility profiles of the corresponding adult populations tested in complementary surveys [33, 34, 38], we could establish that any agrochemical killing less than 50% of wild-caught larvae after 24 h and allowing more than 5% emergence within 6 days is likely to have limited efficacy against *Anopheles* adult populations. Precisely, larval and adult susceptibility to neonicotinoids follow similar trends. Our results showed that larval populations whose adults were resistant to a neonicotinoid (i.e., mortality against the discriminating dose < 90%) typically displayed between 3 and 45% mortality within 24 h and 7–60% emergence after 6 days in water containing a lethal concentration of the active ingredient [33, 34, 38]. Testing a larger number of insecticides on diverse *Anopheles* species would provide more robust guidelines for interpreting mortality, growth, and emergence of larvae reared in pesticide-laced water.

Exposure of non-target insect species to sublethal doses of insecticides affects functions such as motility, behavior, growth, fecundity, and survival [27, 53, 56–59]. According to surveys conducted worldwide, the concentrations of neonicotinoids tested in the present study fell in the upper limits of average values that have previously been detected in contaminated waters and soils [26–28, 60]. Information on the level of contamination in agricultural areas such as Nkolondom is lacking, but neonicotinoids are extensively used for crop protection in this village, and mosquito larvae are presumably chronically exposed to pesticide residues [33]. Tomé et al. [53] showed that at concentrations varying from 0.1 to 15 particles per million (ppm), the insecticides azadirachtin, deltamethrin, imidacloprid, and spinosad displayed concentration-dependent effects on survival and motility in larvae and pupae from a laboratory strain of the yellow fever mosquito *Ae. aegypti*. Compared to *Aedes* mosquitoes, the *An. gambiae* Kisumu strain we tested was susceptible to lower insecticide doses, since 100% larval mortality was reached within 24 h with concentrations ranging from 0.015 to 1.5 ppm. However, field-collected larvae of *An. gambiae* and *An. coluzzii* exhibit varying degrees of adaptation to the sublethal effects of exposure to neonicotinoids and deltamethrin in aquatic habitats. In a recent study, Wu et al. [59] revealed that survival rate decreased by 51.4%, 60.7%, and 48.6%, respectively, when F0 generations of the invasive pest *Spodoptera frugiperda* were exposed to sublethal concentrations (LC_30_) of chlorantraniliprole, dinotefuran, and beta-cypermethrin. In the present study, an approximately 50% survival rate was observed in *An. gambiae* larvae exposed to a lethal dose (LC_99_) of acetamiprid or imidacloprid. Similarly, in contrast to *An. coluzzii*, the pupation rate was not strongly impaired in *An. gambiae* larvae reared in a lethal concentration of neonicotinoids. For example, 70% of L3 *An. gambiae* achieved the pupal stage within 6 days in 0.15 ppm of acetamiprid. In comparison, a pupation rate of 70–80% has been observed in juveniles of *Plutella xylostella* exposed to a significantly lower dose (LC_20_) of emamectin benzoate [58]. Afza et al. [61] revealed that six synthetic insecticides (imidacloprid, thiamethoxam, lambda-cyhalothrin, cypermethrin, chlorpyrifos, and profenofos) at their sublethal doses (LC_30_) suppressed the emergence of adults of *Coccinella septempunctata*, whereas in our study, between 7 and 60% of *An. gambiae* larvae emerged in lethal concentrations of neonicotinoids.

Residual pesticide exposure is an important driver of resistance to public health insecticides [32, 62–67]. It has been hypothesized that resistance is selected in larval breeding sites where immature stages are unintentionally exposed to pesticide residues, and that this tolerance is expressed in adult populations [8]. We conducted a laboratory experiment to assess larval susceptibility, which provides key information on the selective processes that likely lead to the emergence of *Anopheles* larval populations with increased tolerance to some agrochemicals. In our study, mortality rates and life table parameters in wild larvae reared in artificial media containing a lethal concentration of the active ingredient revealed gradients of tolerance, likely reflecting past exposure to pesticide residues in nature. Based on mortality, survival, growth, pupation, and emergence rates, the least effective insecticides against *An. gambiae* larvae were acetamiprid and imidacloprid, two widely used neonicotinoids known to be highly persistent in soil and water [24, 28, 57, 68]. This result suggests that residual pesticide exposure likely plays a key role in *Anopheles* larval resistance to neonicotinoids. This hypothesis has been supported by a laboratory experiment demonstrating that exposure of *Anopheles* larvae to sublethal concentrations of a mixture containing several herbicides, pesticides, and fungicides resulted in ~ 2.5 increase in tolerance to clothianidin [69]. Moreover, neonicotinoid resistance in *An. gambiae* and *An. coluzzii* adults is strongest in agricultural areas, suggesting a correlation between the use of agricultural neonicotinoids and resistance development. Expectedly, chlorfenapyr, which is unlikely to be a residual contaminant in breeding sites and has yet to be extensively used in public vector control programs, was highly toxic to *An. gambiae* and *An. coluzzii* larvae from Yaoundé. Deltamethrin, a pyrethroid widely used for crop protection in Cameroon, revealed low short-term larvicidal activity, but reduced survival within 7 days more effectively than acetamiprid and imidacloprid. Clothianidin also significantly inhibited growth, pupation, and emergence compared to acetamiprid and imidacloprid, although 7% of *An. gambiae* larvae from the farm emerged within 7 days. This pattern is consistent with cross-resistance conferred by residual exposure to neonicotinoids used for crop protection and/or by some preselected metabolic resistance enzymes. However, the sample size of our study is small, and further surveys across a large geographical area are needed to further investigate the correlation between pesticide residues and the development of resistance to neonicotinoids.

Larvae of the sibling species *An. gambiae* and *An. coluzzii* displayed similar levels of susceptibility to chlorfenapyr and to deltamethrin but exhibited striking variation in tolerance to neonicotinoids. This result reflects susceptibility profiles described in adult populations from Yaoundé: *An. coluzzii* adults are generally susceptible to neonicotinoids, while *An. gambiae* is resistant to at least four different active ingredients [33, 34, 38]. Variations have been observed in the level of resistance to pyrethroids as well as the frequency of some resistance alleles between *An. gambiae* and *An. coluzzii* from Yaoundé [70–72]. The intensity of overexpression of some CYPs and GSTs also differs between *An. gambiae* and *An. coluzzii* from this area [47]. Some of these enzymes could be involved in metabolic resistance to neonicotinoids and could contribute to the observed variation in susceptibility among larvae and adults of both species. On the other hand, in addition to potential specie-specific metabolic resistance, varying levels of selection pressure in their habitats may also explain the difference between *An. gambiae* and *An. coluzzii*. Notably, agricultural practices associated with the spaying of neonicotinoids are more prevalent in the countryside, and therefore *An. gambiae* may be more exposed to neonicotinoid residues than urban *An. coluzzii* mosquitoes.

Although our findings are based on only two *Anopheles* species sampled from a relatively small geographical area, the results highlight levels of resistance that could be an obstacle to the use of neonicotinoids for malaria prevention [2, 32–34, 42]. Moreover, it has been shown that neonicotinoid-resistant adult mosquitoes from the Nkolondom farm display reduce susceptibility to SumiShield^®^ 50WG, a clothianidin formulation prequalified for indoor residual spraying [33]. Future research directions include testing immature stages from different *Anopheles* species while evaluating their degree of exposure to neonicotinoid residues in larval habitats. A better understanding of the role of some detoxification enzymes will also provide critical insights into the cross-resistance mechanisms contributing to the emergence of neonicotinoid resistance in *Anopheles* mosquitoes.

## Conclusions

Repurposing of agrochemicals has thus far provided a rapid mechanism for identifying new candidate insecticides used for malaria prevention. The example of neonicotinoids emphasizes the crucial role of evaluating susceptibility and cross-resistance in larval populations. Such information could be complementary to routine adult susceptibility testing based on standard mortality-based bioassays. Combining both approaches may offer a more robust framework to better evaluate preexisting levels of adaptation to agrochemicals used for malaria prevention.

## Data Availability

The data for this study have been presented within this article.
